# Genome-Wide Transcription Analysis of Electroacupuncture Precondition-Induced Ischemic Tolerance on SD Rat With Ischemia–Reperfusion Injury

**DOI:** 10.3389/fgene.2021.719201

**Published:** 2021-08-16

**Authors:** Shuping Fu, Meiling Yu, Houxi Xu, Qing Liu, Xiaoxiao Li, Yaling Wang, Yonglin Chen, Lingling Meng, Yiting Qiu, Xinyue Jing, Chenchen Liu, Shengfeng Lu

**Affiliations:** ^1^Key Laboratory of Acupuncture and Medicine Research of Ministry of Education, Nanjing University of Chinese Medicine, Nanjing, China; ^2^School of Basic Medical Sciences, Shanghai University of Traditional Chinese Medicine, Shanghai, China; ^3^School of Nursing, Nanjing University of Chinese Medicine, Nanjing, China

**Keywords:** electroacupuncture, precondition, ischemic stroke, ischemic tolerance, RNA-Seq

## Abstract

Acupuncture promotes the recovery of neurological function by the overall improvement of ischemic brain injury. It is not only regarded as a rehabilitative treatment but also a pretreatment method for stroke. However, its mechanism has not been fully elucidated. In this study, rats were treated with electroacupuncture (EA) at Baihui (GV20) for 30 min/day for 6 days, ahead of conducting cerebral ischemia–reperfusion (I/R) injury. Infarction volume, Evans blue leakage, and neurological deficits were evaluated at 24 h after I/R injury. Then, the ipsilateral ischemic brain was isolated for RNA sequencing (RNA-Seq) to identify molecular consequences. The results showed that EA pretreatment decreased blood–brain barrier (BBB) permeability, reduced brain infarction volume, and improved neurological outcomes. EA pretreatment could upregulate expression of antivirus and immunity activity-associated genes (such as *Ifit1*, *Ifit3*, *Irf7*, and *Oasla*) and downregulate expression of matrix disruption-associated genes (*Col24a1*, *Col11a1*, *Col27a1*, etc.) in healthy rats. In addition, it could partially reverse or ameliorate genome-wide transcription changes of the ipsilateral ischemic brain. For the first time, this study provides insight into genomic network modulation of a healthy rat with EA treatment and a EA-preconditioned rat under subsequent I/R injury, which is helpful in explaining acupuncture precondition-induced ischemic tolerance of stroke. It also provides new strategies and targets for the prevention of ischemic stroke.

## Introduction

Stroke is one of the most common causes of death and adult disability worldwide, bringing a significant burden to families and society (Mukherjee and Patil, 2011). Therefore, preventing stroke, reducing mortality, and preventing post-stroke injury have always been the focus of neurological researchers. The main preventive measures in clinical practice are improving lifestyle and actively controlling stroke risk factors such as obesity, diabetes, hypertension, hyperlipidemia, and coronary heart disease. However, since Murry et al. (1986) proposed that exposure to sublethal or non-injurious stimuli can increase resistance to the subsequent, prolonged, and lethal cell injury in ischemic myocardium, precondition-induced ischemic tolerance has provided a new perspective in stroke preventive strategies. Various kinds of precondition measures have been applied in stroke research, such as regional or remote ischemia (Perez-Pinzon et al., 1997; Zhao et al., 2004; Plamondon et al., 2008; Yang et al., 2020) and pharmacological precondition (e.g., statin, melatonin, and Tocovid) (Jiao et al., 2018; Dong et al., 2019; Liu et al., 2019). Accumulating preclinical evidence has demonstrated that these methods could induce neuroprotection against brain ischemia–reperfusion (I/R) injury. However, due to the invasion of ischemia approaches or rigorous adaption of drugs itself, not all methods mentioned above can directly be applied in clinical settings (Li and Wang, 2013; Sharma et al., 2020). It is necessary to find more clinically feasible approaches for the prevention of stroke.

Acupuncture has been applied for more than 3,000 years as a treatment for many diseases. It is recommended by the World Health Organization (WHO) as an alternative and complementary therapy for stroke; both clinical trials and preclinical studies demonstrated the neuroprotective efficacy of acupuncture in post-stroke rehabilitation (Li and Wang, 2013; Chavez et al., 2017). It is also regarded as a promising preventive strategy for stroke due to the increasing evidence showing that acupuncture precondition could induce ischemic tolerance to cerebral ischemia in rats. Xiong et al. (2003) first reported the neuroprotective effect of electroacupuncture (EA) pretreatment on ischemic stroke rats. They applied EA preconditioning at the Baihui acupoint (GV20) 30 min/day for 5 days on Sprague Dawley (SD) rats. Twenty-four hours after the last treatment, transient middle artery occlusion (MCAO) was performed. The results showed that EA precondition could significantly attenuate the infarction volume, brain edema, and blood–brain barrier (BBB) disruption and improve neurological function. These effects were associated with decreased MMP-9 activity, increased CB1R-mediated phosphorylation of GSK-3β, and activation of the canonical Notch pathway (Dong et al., 2009; Zhao et al., 2012; Wei et al., 2014). Zou et al. (2015) confirmed that EA pretreatment significantly reduced BBB permeability and brain edema, which was related to the alleviation of the degradation of tight junction proteins and inhibition of the expression of p-caveolin-1 in the endothelial cells. Wang et al. (2012) and Long et al. (2019) reported that EA pretreatment had sound effects on antioxidant stress, anti-inflammation, and neuroprotection. However, these studies were mainly focused on exploring the post-stroke molecular changes of acupuncture precondition. Few researchers are concerned about what happened in the healthy brain with acupuncture treatment and the effect of these changes on the subsequent ischemic stroke. It should be an essential and indispensable part of the anti-ischemic injury effect of acupuncture precondition.

We aimed to further clarify the underlying mechanism of acupuncture precondition-induced ischemic tolerance on stroke in this study, after confirming the EA precondition’s anti-ischemic injury effect at Baihui (GV20) on I/R rats. For the first time, we employed high-throughput messenger RNA sequencing (RNA-Seq) to study the genome-wide response of transcriptomes in healthy rats with EA treatment and EA-preconditioned rats under subsequent I/R injury. Our data suggested that EA precondition could activate immune response-associated gene expression in healthy rat brains and partially reverse or ameliorate genome-wide transcription changes of the ipsilateral ischemic brain induced by I/R injury. It is helpful to explain the acupuncture precondition-induced ischemic tolerance of stroke.

## Materials and Methods

### Animals and Grouping

**FIGURE 1:**
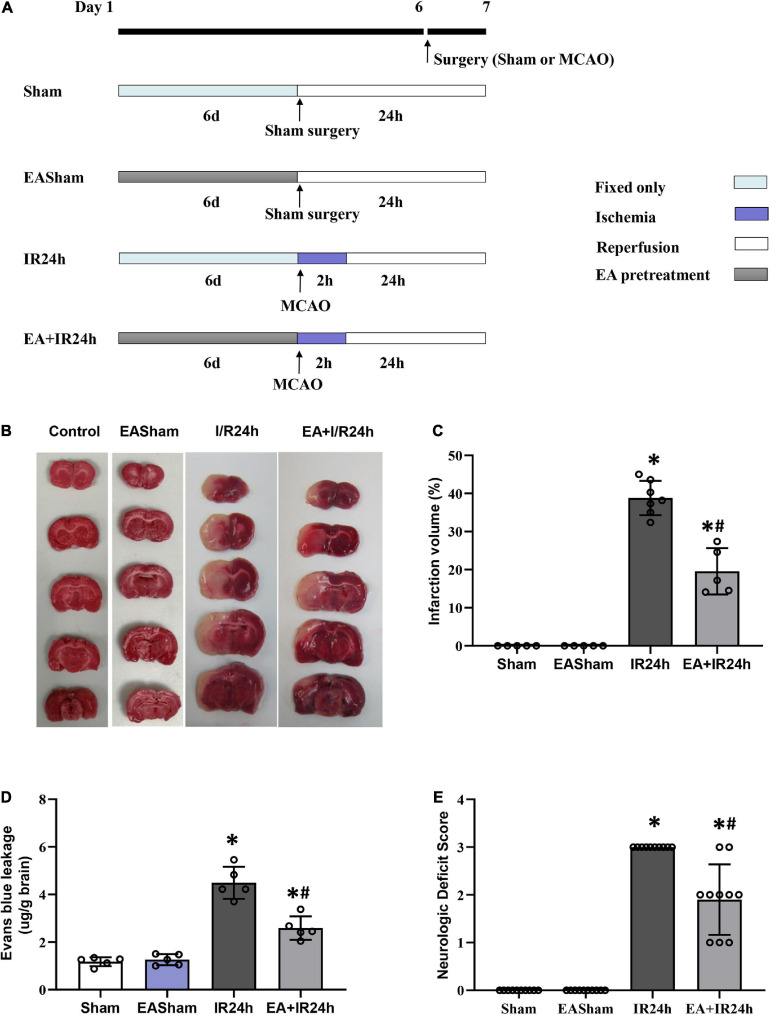
Electroacupuncture (EA) pretreatment significantly ameliorated I/R injury of Sprague Dawley (SD) rats. **(A)** Animal group and experiment protocol. All male adult SD rats were randomly divided into four groups: Sham surgery group (Sham), 24 h post-I/R group (IR24h), EA pretreated plus Sham surgery group (EASham), and EA pretreated plus 24 h post-I/R group (EA + IR24h). In brief, cerebral I/R injury was induced by middle artery occlusion (MCAO) experiments. For the Sham group, the rats underwent a similar surgical procedure without MCA occlusion. In the EA pretreatment groups, rats were pretreated with EA at GV20 acupoint for 30 min/day for 6 consecutive days. The rats in the EA + IR24h group were applied MCAO surgery at 2 h after finished the sixth EA pretreatment. The rats in the EASham group were used a similar surgical procedure without MCA occlusion at 2 h after finished the sixth EA pretreatment. **(B)** Representative image of TTC staining. **(C)** Quantitation results of TTC staining. The ischemic area was evaluated by calculating the hemispheric lesion area with Image J software, and the relative infarction volume percentage was used to represent as a bar graph. **(D)** Blood–brain barrier (BBB) Permeability in each group was determined by Evans blue extravasation assay. **(E)** Neurologic function score was evaluated by Bederson’s neurologic deficit examination grading system. All data were represented as Mean ± SD, *n* = 5–10, *P < 0.01 compared to the Sham group, ^#^*P* < 0.01 compared to the I/R 24 h group.

Male adult SD rats (250–300 g), supplied by the Experimental Animal Center of the Nanjing University of Chinese Medicine, were used for this study. All rats were housed at 23 ± 1°C in a 12-h light/dark cycle with free access to water and food. The rats were fasted (with free access to water) for 12 h before surgery. As Figure 1A showed, all rats were randomly divided into four groups: sham surgery group (Sham), 24 h post-I/R group (IR24h), EA-pretreated plus sham surgery group (EASham), and EA-pretreated plus 24 h post-I/R group (EA + IR24h). The study was approved by the Institutional Animal Care and Use Committee of the Nanjing University of Chinese Medicine.

### MCAO

Rats were subjected to MCAO experiments to induce cerebral I/R injury as our previously reported protocol (Fu et al., 2014). In brief, rats were anesthetized by inhalation of 5% isoflurane and maintained with 2% isoflurane in a mixture of 70% N_2_O and 30% O_2_. A 3/0 monofilament nylon suture (Surgical Specialties & Look, MA, Mexico) was inserted into the left external carotid artery, advancing to the anterior cerebral artery through the internal carotid artery to occlude the middle cerebral artery at its origin to induce ischemia. After 2 h of MCAO occlusion, the intraluminal suture was withdrawn to induce reperfusion. In 2 h of MCAO ischemia after surgery and subsequent reperfusion, the rats were transferred to an intensive care incubator. The temperature was kept at 37°C until the animals woke up completely. For the sham group, the rats underwent the same surgical procedure without suture insertion.

### EA Treatment

According to the previous study (Xiong et al., 2003; Wang et al., 2005), the Baihui acupoint (GV20), located at the midpoint of the connecting line between the auricular apices, was selected for EA preconditioning. The rats were pretreated with EA at the GV20 acupoint for 30 min/day for 6 consecutive days, with parameters of 2/15 Hz at an intensity level of 1 mA, which was stimulated by an electrical stimulator with a stimulus isolation unit (Han Acuten, WQ1002F, Beijing, China). As shown in Figure 1A, the rats in the EA + IR24h group underwent MCAO surgery at 2 h after the sixth EA pretreatment finished. The rats in the EASham group underwent the same surgical procedure without the insertion of a suture at 2 h after completion of the sixth EA pretreatment.

### Measurement of Infarct Volume

After 2 h of ischemia and subsequent 24 h of reperfusion, rat brains were isolated and frozen at –20°C for 30 min. Then the brains were sliced into coronal sections in a brain groove (2 mm thick), followed by staining with 2,3,5-triphenyl tetrazolium chloride (TTC, Sigma) for 20 min at room temperature. The infarct area was evaluated by calculating the hemispheric lesion area with ImageJ software (ImageJ, MD, United States). The relative infarction volume percentage (RIVP) was calculated as RIVP = IVA/TA × 100%. IVA and TA were the total infarcted area and the total area of five coronal sections, respectively.

### Evaluation of BBB Permeability

The BBB permeability was evaluated by measuring the extravasation of Evans blue as our previously reported protocol (Fu et al., 2014). Briefly, 2% Evans blue solution was intravenously injected (2 ml/kg, Sigma) at 1 h before sacrifice. After deeply anesthetizing with pentobarbital (50 mg/kg, Sigma), the rats were perfused with saline through the left ventricle until the colorless fluid was obtained from the right atrium. The ipsilateral ischemic cortex was removed and incubated in *N*,*N*′-dimethylformamide (5 L/kg brain tissue, Sigma) in a water bath with a temperature of 60°C for 24 h. Evans blue content in supernatants was determined at 632 nM using a microplate reader (Synergy H1, BioTek, United States). Gradient concentrations of Evans blue were used to build a standard curve to quantify the Evans blue retention in the hemisphere.

### Neurologic Function Assay

The neurologic function was evaluated by Bederson’s neurologic examination grading system (Bederson et al., 1986). In brief, rats were held by the tail, and normal rats that extended both forelimbs forward and had no neurologic deficit were assigned grade 0. Rats with consistent forelimb flexion and had no other abnormality were given grade 1. Rats with decreased resistance to lateral push, constant forelimb flexion, and no circling were assigned grade 2. Rats with the same behavior as grade 2 and with circling were given grade 3.

### RNA-Seq and Computational Analysis for RNA-Seq Data

Total RNA was extracted by TRIzol reagent (Invitrogen, Cat No. 15596018) from the ipsilateral ischemic brain. RNA concentration was quantified by Qubit1 2.0 fluorometer (Invitrogen) with Qubit RNA BR assay kit (Invitrogen, Cat No. Q10211). According to the manufacturer’s protocols, RNA quality control was performed using Agilent 2100 Bio-analyzer (Agilent Technologies, Inc., CA, United States). For RNA-Seq, RNA samples were prepared according to the TruSeq RNA Sample Preparation v2 protocol, 3 replicates for each group, a total of 12 samples. The DNA libraries were applied to the cluster generation and sequencing using the cBOT Multiplex re-hybridization plate and TruSeq SBS kit V3. Sequencing was performed using Illumina HiSeq 2000 (Illumina, CA, United States). After sequencing, raw fastq files were extracted from Illumina BCL using the Illumina CASAVA program. Primary component analysis (PCA) was applied by R software. The single-end reads of biological triplicates obtained from each sample were aligned to the rat reference genome (UCSC rn4 assembly) using the TopHat program. The DESeq2 program was used to assemble individual transcripts from RNA-Seq reads aligned to the genome and qualify the expression level of each transcript. Differential transcript expression analysis was performed with the Cuffdiff program. The gene’s functional annotation and pathway were analyzed using the clusterProfiler and DAVID Bioinformatics Resources (Huang et al., 2009a,b). The protein–protein interaction (PPI) internet analysis was performed with STRING v11 (Szklarczyk et al., 2019). Only genes that exhibited changes in expression of more than twofold [|log2(FC)| > 1] and had *P-*values adjusted using the Benjamini-Hochberg procedure of less than 0.05 (*Padj* < 0.05) were subjected to bioinformatic analysis.

### Real-Time RT-PCR Assay and Statistics

Total RNA was extracted by TRIzol (Invitrogen), and 4 μg of RNA was reversed to cDNA according to the manufacturer’s instructions (#1621, Thermo Fisher Scientific, Waltham, MA, United States). The primer sequences are shown in Supplementary Table 9. For real-time quantitative PCR analysis, the cDNA samples were amplified with triplication using SYBR Green (Thermo Fisher Scientific, #PC4602) with 200 nM of gene-specific primers and run on the ViiA^TM^ 7 real-time PCR systems (ViiA^TM^ 7, Applied Biosystems, CA, United States) using the manufacturer’s protocol. Data were analyzed by the threshold cycle (Ct) relative quantification method. Statistically significant differences were considered with *P* < 0.05.

### Statistical Analysis

Data were presented as means ± standard deviation (SD). Statistical analysis was performed using SPSS 19.0 (SPSS, Chicago, IL, United States). A one-way analysis of variance (ANOVA) for multiple group comparisons followed by Dunnett’s test for two-group comparisons within the multiple groups was performed; *P* < 0.05 was considered statistically significant.

## Results

### EA Pretreatment Significantly Ameliorated I/R Injury of SD Rats

We firstly examined the neuroprotective effect of EA pretreatment on I/R injury in SD rats. The infarct volume of the I/R injury brain was detected by TTC staining. As shown in Figures 1B,C, the infarct size in the ipsilateral hemisphere of the IR24h group rises up to 38.9 ± 3.93%, whereas the EA + IR24h group had a dramatically reduced infarct volume (19.5 ± 4.87%). BBB hyperpermeability, which mainly contributed to infarction volume, brain edema, and hemorrhage, is a vital brain damage marker of cerebral I/R injury. We performed Evans blue extravasation assay to evaluate the protective effect of EA pretreatment on BBB integrity in I/R rats. The results showed that the Evans blue leakage of the rats in the EASham group had no difference from that of the Sham group. However, the rats in the IR24h group had higher BBB permeability of up to 4.5 ± 0.67 μg/mg. In contrast, the EA + IR24h group had 44% lower BBB permeability when compared to the IR24h group (Figure 1D). In addition, we evaluated the effects of EA pretreatment on functional recovery based on behavior deficit, which is a major endpoint in clinical trials. The rats of the EA + IR24h group displayed much improved neurological behavior deficit scores than the IR24h group (Figure 1E). These results suggest that EA pretreatment exerted neuroprotective effects against cerebral I/R injury.

### EA Pretreatment Upregulated Antiviral and Immunity Activity-Related Genes of the Healthy SD Rats

**FIGURE 2:**
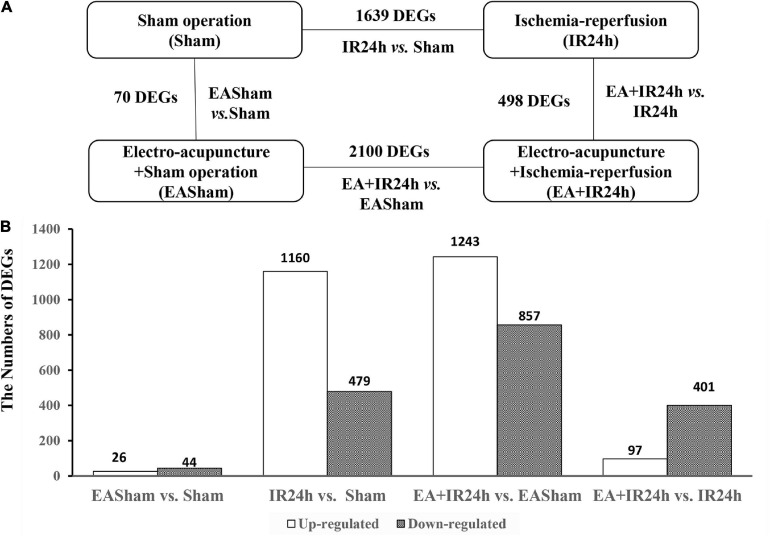
Characterization of differentially expressed genes (DEGs) in Sprague Dawley (SD) rats with electroacupuncture (EA) pretreatment or/and I/R injury. **(A)** Schematic comparison for groups DEGs results in EASham vs. Sham, IR24h vs. Sham, EA + IR24h vs. EASham, EA + IR24h vs. IR24h. **(B)** The number of up-and down-regulated DEGs in each comparative group. The numbers of DEGs were placed above the bars, and white bar means up-regulated DEGs, black bar means down-regulated DEGs. Genes with a change in mRNA expression more than twofold [|log2(FC)| > 1] compared with the baseline value and with a Padj value less than 0.05 (*Padj* < 0.05) were selected for analysis.

**FIGURE 3:**
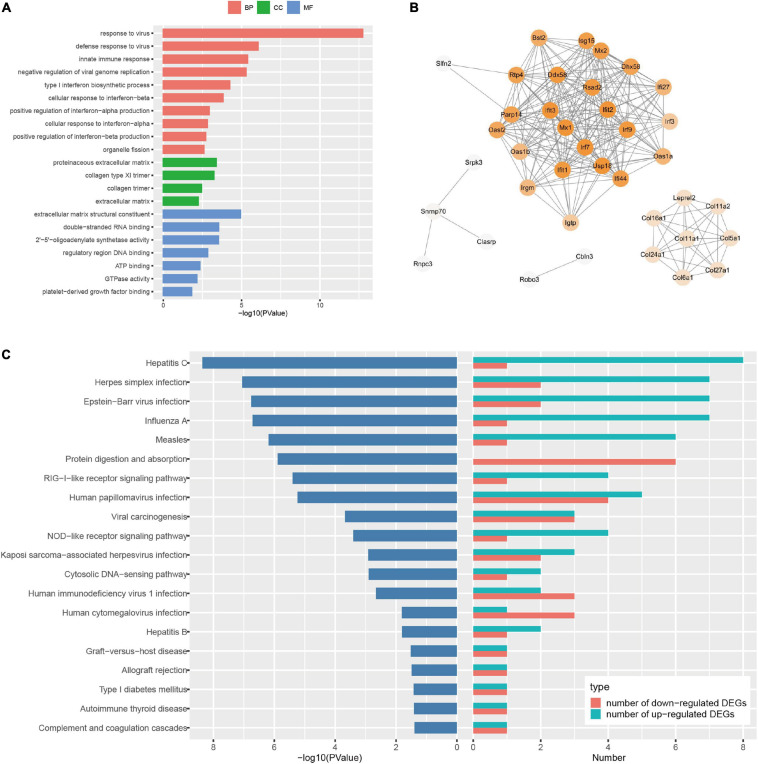
Bioinformatic analysis of the differentially expressed genes (DEGs) in Sprague Dawley (SD) rats with electroacupuncture (EA) pretreatment. **(A)** Top 10 categories of GO analysis results. *P* < 0.05. **(B)** Protein-protein interaction (PPI) internet analysis of DEGs in EASham vs. Sham. **(C)** Top 20 categories of Kyoto Encyclopedia of Genes and Genomes (KEGG) signaling pathway and corresponding *P*-values, as well as the number of up- and down- regulated DEGs in EASham vs. Sham.

To uncover the potential mechanism of EA pretreatment on healthy SD rats, the sham-operated animals were used as a control to analyze differentially expressed genes (DEGs) in healthy rat brains after EA pretreatment. Seventy DEGs with |log2(FC)| > 1 and *Padj* < 0.05 were identified in EASham vs. Sham (Figure 2A), in which 26 (37.1%) genes (*Ifit*, *Ifit2*, *Ifit3*, *Mx1*, *Mx2*, *Oasla*, and other genes) were upregulated, while 44 (62.9%) genes (*Col24a1*, *Col11a1*, *Col27a1*, *Irf3*, *Hdac10*, and other genes) were downregulated (Figure 2B and Supplementary Table 1). Gene ontology (GO) annotation indicated that these DEGs were predominantly expressed in the proteinaceous extracellular matrix, collagen type XI trimer, collagen trimers, and extracellular matrix components. According to the molecular functions of their encoded proteins, most of these DEGs were involved in extracellular matrix structural constituent, double-stranded RNA binding, ATP binding, regulatory region DNA binding, and so on. The biological processes associated with these DEGs were divided into 18 functional categories. The top five categories included the defense response to the virus process, innate immune response, negative regulation of the viral genome replication process, and type I interferon (IFN) biosynthetic process (Figure 3A). The functional annotation results of the proteins encoded by these DEGs revealed that most DEGs belonged to the categories of innate immunity/immunity, antiviral defense, extracellular matrix, collagen, nucleotide binding, and so on (Supplementary Table 2). The Kyoto Encyclopedia of Genes and Genomes (KEGG) pathway analysis confirmed that the DEGs were significantly enriched in 20 signaling pathways, and most of them were predominantly involved in viral- and immunity response-related diseases and signaling pathways, such as hepatitis C, Epstein–Barr virus infection, viral carcinogenesis, human immunodeficiency virus 1 infection, RIG-I-like receptor signaling pathway, and human papillomavirus infection (Figure 3C). Combining with the PPI network analysis, there were tight interaction relationships in these EA-pretreatment-induced DEGs. All upregulated DEGs, including *ifit1*, *isg15*, *dhx58*, *irf9*, *oals1a*, and *mx2*, were enriched in one cluster. And most of them were mainly involved in protective biological processes, such as defense response to the virus, innate immunity response, negative regulation of viral genome replication, mitochondrial fission, and response to IFN-beta. Concomitantly, some of the EA-pretreatment-downregulated DEGs, such as *Col6a1*, *Col5a1*, *Col27a1*, *Leprel2*, and *Col24a1*, formed another cluster contributing to protein digestion and absorption response to inflammation reaction (Figure 3B).

### The Characteristic of I/R Injury-Induced Transcriptome Profiles of the Healthy and EA-Pretreated SD Rat Brain

**FIGURE 4:**
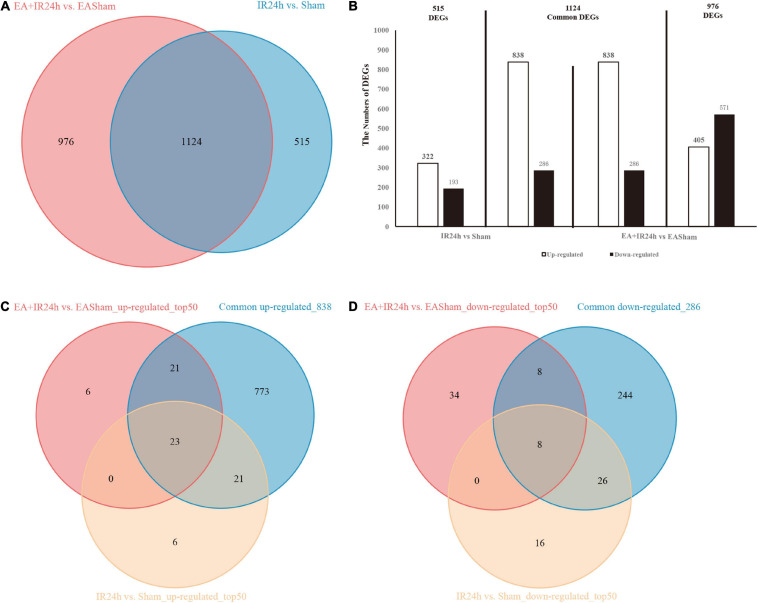
Differentially expressed genes (DEGs) comparison between normal and EA-pretreatment Sprague Dawley (SD) rats under I/R condition. **(A)** The numbers of DEGs overlapped in IR24h vs. Sham and EA + IR24h vs. EASham. **(B)** Diagrams showing DEGs that lie within the section of sets on the Venn diagrams. The numbers of DEGs were placed above the bars, and white bar means up-regulated DEGs, black bar means down-regulated DEGs. **(C)** The number of DEGs overlapped in common 838 and top 50 up-regulated genes in IR24h vs. Sham and EA + IR24h vs. EASham. **(D)** The number of DEGs coincided in common 286 and the top 50 down-regulated genes in IR24h vs. Sham and EA + IR24h vs. EASham.

To explore the genome-wide transcriptome response of rat brains to I/R injury subject to EA pretreatment or non-pretreatment, as shown in Figure 2A, sham-operated and EA-pretreated plus sham-operated animals were taken as controls to the IR24h group and the EA + IR24h group animals, respectively (IR24h vs. Sham and EA + IR24h vs. EASham). In the total DEGs of each comparative group, both groups had more upregulated genes than downregulated genes (Figure 2B), so did the 1,124 overlapped genes of these 2 groups (Figures 4A,B). However, the expression patterns for the non-common regulated DEGs in these 2 groups were opposite. In the IR24h group, there were more upregulated DEGs than downregulated DEGs, but in the EA + IR24h group, there were less upregulated DEGs than downregulated DEGs (Figure 4B). Comparing the top 50 upregulated DEGs, we found that 44 genes in each group were the overlapped upregulated DEGs of these 2 groups. And 23 genes (*Sfn*, *Cxcl1*, *Ccl4*, *Il11*, *Mmp12*, and so on) in these 2 comparative groups were identical (Figure 4C). It was worth noting that, except *sfn*, the expression fold change of these 23 common genes in the IR24h group was obviously greater than the EA + IR24h group (Supplementary Table 3). For the top 50 downregulated DEGs, 68% DEGs (34/50) in the IR24h group and 32% DEGs (16/50) in the EA + IR24h group were the overlapped downregulated DEGs of these 2 comparative groups (Figure 4D). But there were only 8 identical genes in the top 50 DEGs of these 2 groups, which are *Gucy1a3*, *Olr59*, *Tacr3*, *Srpk3*, *Sptlc3*, *Tph1*, *Pdc*, and *Mpp4*. Apart from *Sptlc3* and *Olr59*, fold changes of the other six common gene expressions in the IR24h group were all lesser than that in the EA + IR24h group (Supplementary Table 3).

**FIGURE 5:**
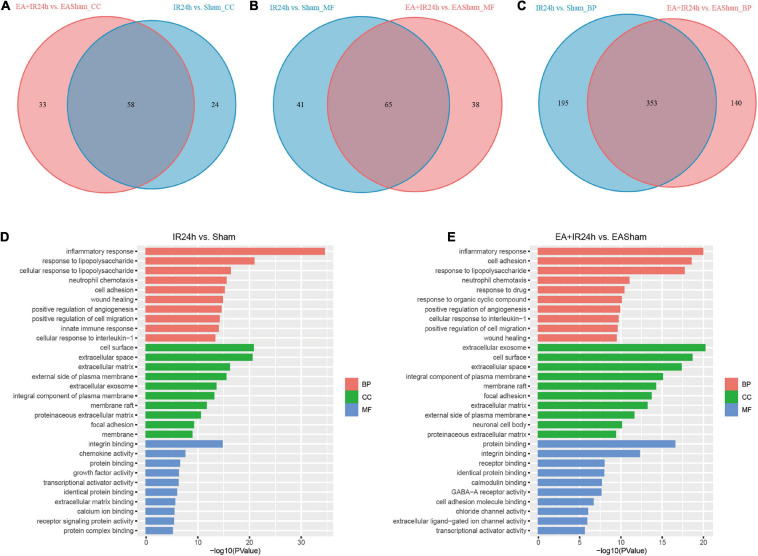
Gene ontology (GO) analysis of the DEGs in normal and EA-pretreatment Sprague Dawley (SD) rats under I/R condition. Venn diagram showing common and unique numbers of cell component (CC) categories **(A)** molecular function (MF) categories **(B)** and biological process (BP) categories **(C)** associated with DEGs in IR24h vs. Sham and EA + IR24h vs. EASham. Top 10 GO categories in IR24h vs. Sham **(D)** and EA + IR24h vs. EAsham **(E)** and corresponding –log10 (*P*-value).

To further investigate the possible mechanism of the similarities and differences of pathological changes in EA pretreatment and non-pretreatment in SD rat subjected to I/R injury, bioinformatic analysis was applied. GO annotation analysis revealed that the DEGs in IR24h vs. Sham and EA + IR24h vs. EASham were widely distributed in 82 and 91 cell components, respectively, in which 58 cell components were overlapped. The top five categories were cytoplasm, plasma membrane, extracellular exosome, membrane, and extracellular space (Figures 5A,D). In addition, the top five categories of the unique cell components in the IR24h group were death-inducing signaling complex, extrinsic component of cytoplasmic side of the plasma membrane, transcription factor complex, MHC class II protein complex, and vesicle, while in the EA + IR24h group, they were GABA-A receptor complex, chloride channel complex, synapse, presynaptic membrane, and neuronal cell body membrane (Supplementary Table 4).

The molecular function enrichment results revealed that there were 65 common categories in these 2 groups, 9 of the top 10 in IR24h vs. Sham and 7 of the top 10 categories in EA + IR24h vs. EASham belong to the common categories, such as integrin binding, chemokine activity, protein binding, and transcriptional activator activity. DEGs of the IR24h group also exclusively had receptor signaling protein activity, regulatory region DNA binding, CCR2 chemokine receptor binding, and interleukin-1 receptor binding activity, etc. The EA + IR24h group DEGs are exclusively related to the molecular functions of GABA-A receptor activity, chloride channel activity, extracellular ligand-gated ion channel activity, and so on (Figures 5B,D,E and Supplementary Table 5). Biological process analysis revealed that DEGs in the IR24h group were divided into 548 categories vs. 493 categories in the EA + IR24h group, and 353 categories were the same. In accord with this ratio, 8 of the top 10 categories of these 2 comparative groups were identical, including inflammatory response, neutrophil chemotaxis, positive regulation of angiogenesis and cell migration, and cell adhesion (Figures 5C–E). The functional annotation of DEGs was identified using the DAVID program. A total of 53 significant categories were found in these 2 groups, and 26 functional categories, including glycoprotein, disulfide bond, immunity, inflammatory response, and apoptosis, were the same in non-pretreated and EA-pretreated rat response to I/R injury. Meanwhile, the numbers of upregulated DEGs in the most common categories were similar. Still, the numbers of downregulated DEGs in the EA-pretreated rats were more than that in the non-pretreated rats. In addition, four exclusive categories, namely, proteoglycan, sushi, angiogenesis, and leucine-rich repeat, were identified in the IR24h group. And in the EA + IR24h group, there were 23 exclusive categories: ion channel, ion transport, postsynaptic cell membrane, cell junction, etc. (Table 1).

**TABLE 1:** Analysis of functional categories of the proteins encoded by differentially expressed genes (DEGs).

**Functional categories**	**IR24h vs. Sham**				**EA + IR24h vs. EAsham**			
	**Up**	**Down**	**Count**	**Padj**	**Up**	**Down**	**Count**	**Padj**
Glycoprotein	230	98	328	9.11E-37	224	194	418	1.38E-48
Disulfide bond	259	76	335	2.15E-36	261	154	415	3.13E-43
Signal	341	92	433	7.45E-29	334	181	515	6.34E-27
Immunity	65	3	68	1.78E-21	57	7	64	3.16E-13
Secreted	149	22	173	6.73E-21	157	37	194	2.19E-17
Inflammatory response	33	3	36	2.44E-16	28	2	30	3.97E-08
Innate immunity	40	2	42	4.30E-14	31	4	35	2.94E-06
Phosphoprotein	291	115	406	2.94E-08	340	216	556	1.67E-19
Chemotaxis	20	1	21	6.22E-08	20	4	24	1.15E-08
Extracellular matrix	28	7	35	6.23E-08	28	6	34	1.01E-04
Calcium	56	29	85	4.10E-06	55	50	105	2.55E-07
Cell adhesion	39	11	50	2.09E-05	36	33	69	5.60E-10
EGF-like domain	29	5	34	1.36E-04	28	14	41	2.30E-05
Cytokine	25	1	26	1.55E-04	24	4	28	0.001256
Proteoglycan	13	1	14	1.91E-04				
Immunoglobulin domain	33	10	43	4.88E-04		25	53	5.15E-05
Lipoprotein	41	26	67	5.83E-04	33	41	74	0.021695
Proto-oncogene	15	0	15	6.74E-04	13	2	15	0.011563
Growth factor	22	3	25	0.002506	20	7	27	0.017326
Sushi	10	1	11	0.003817				
Calmodulin-binding	9	14	23	0.004999	12	18	30	7.10E-05
Lectin	22	2	24	0.005994	24	4	28	0.004375
Membrane	415	210	625	0.007314	408	386	794	7.85E-04
Angiogenesis	16	0	16	0.008617				
SH2 domain	16	1	17	0.008839	16	3	19	0.013471
Apoptosis	37	4	41	0.016089	41	12	53	5.27E-04
Palmitate	22	13	35	0.016942	18	24	42	0.009308
Leucine-rich repeat	15	15	30	0.022366				
Cleavage on pair of basic residues	27	3	30	0.0248	29	11	40	2.85E-04
Ubl conjugation	77	13	90	0.032241	82	31	113	0.006292
Ion channel					12	56	68	7.39E-09
Ion transport					19	78	97	6.10E-08
Postsynaptic cell membrane					3	37	40	1.69E-06
Cell junction					20	65	85	4.84E-06
Cytoplasm					217	91	308	1.72E-05
EGF-like domain					27	14	41	2.30E-05
Metal-binding					151	124	275	4.00E-05
Alternative splicing					57	82	139	7.82E-05
Ligand-gated ion channel					1	22	23	9.96E-05
Synapse					3	54	57	2.07E-04
Chloride					4	15	19	2.24E-04
Chloride channel					3	13	16	5.57E-04
Voltage-gated channel					4	27	31	0.001481
Biological rhythms					13	9	22	0.003837
Integrin					12	4	16	0.007916
Kinase					50	41	91	0.008905
Developmental protein					36	40	76	0.01493
Growth factor binding					8	0	8	0.015289
ATP-binding					75	63	138	0.017687
Signal-anchor					26	16	42	0.021811
Symport					5	17	22	0.02705
Nucleotide-binding					90	79	169	0.036916
Adaptive immunity					11	4	15	0.044239

*Associative functional annotation analysis of DEGs for comparison groups is carried out according to the DAVID database. The number of up-regulated (Up) and down-regulated (Down) DEGs of the healthy or 6 days electroacupuncture (EA)-pretreatment Sprague Dawley (SD) rat brains under I/R condition, total DEGs number, as well as the *P*-values adjusted using the Bonferroni procedure (Padj), are shown.*

**FIGURE 6:**
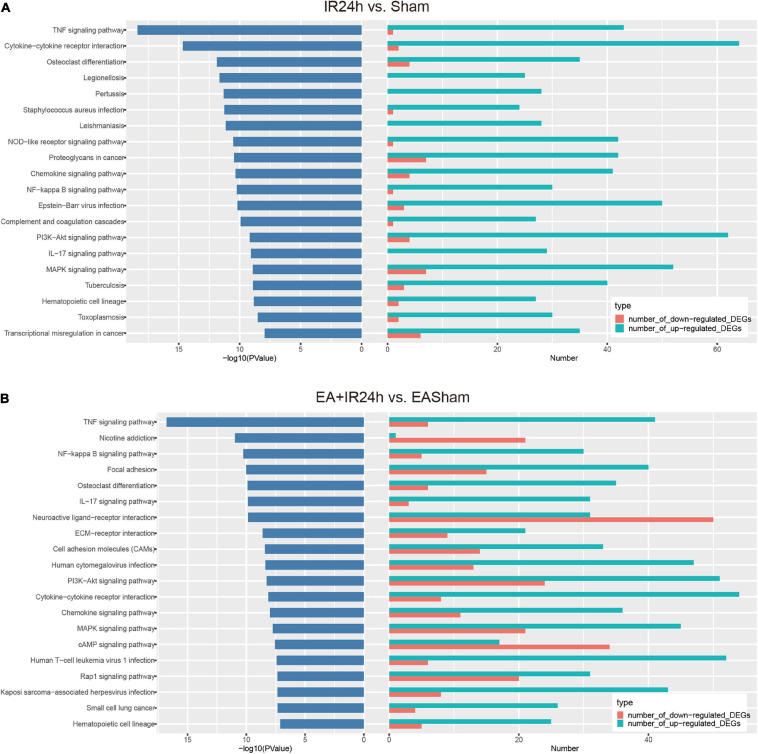
Kyoto Encyclopedia of Genes and Genomes (KEGG) pathway analysis of the differentially expressed genes (DEGs) in I/R injury rats with electroacupuncture (EA) pretreatment and non-pretreatment. Top20 categories of KEGG signaling pathway and corresponding –log10 (*P*-value), as well as the corresponding numbers of up- and down-regulated DEGs in IR24h vs. Sham **(A)** and EA + IR24h vs. EASham **(B)**.

Kyoto Encyclopedia of Genes and Genomes pathway enrichment analysis showed that the total DEGs in IR24h vs. Sham were involved in 131 significantly signaling pathway categories. The upregulated and downregulated DEGs were enriched in 112 and 43 signaling pathways, respectively. And three identical signaling pathways, namely, basal cell carcinoma, inflammatory mediator regulation of TRP channels, and dilated cardiomyopathy, were found among these three subgroups. Over 90% of the upregulated DEG enriched pathways were overlapped with total DEGs, but only 58.1% of the downregulated DEG enriched pathways were overlapped with total DEGs. Furthermore, in the top 20 significantly signaling pathways of total DEGs, 19 of them were overlaid with the top 20 categories of the upregulated DEGs (Supplementary Figure 1). These top 20 categories are predominantly involved in the inflammatory response, such as TNF signaling pathway, cytokine–cytokine receptor interaction, osteoclast differentiation, chemokine signaling pathway, NF-kappa B signaling pathway, and IL-17 signaling pathway (Figure 6A).

In EA + IR24h vs. EASham, the numbers of enriched signaling pathways of the total, upregulated, and downregulated DEGs were 147, 110, and 70, respectively. Of the upregulated DEG enriched pathways, 91.8% overlapped with total DEGs vs. 77.1% of the downregulated DEG enriched pathways. Twelve common categories (Rap1 signaling pathway, PI3K-Akt signaling pathway, MAPK signaling pathway, ECM–receptor interaction, cell adhesion molecules, etc.) were found in these three DEG subgroups (Supplementary Figure 2). In the top 20 categories of total DEGs, 12 categories, including TNF signaling pathway, NF-kappa B signaling pathway, focal adhesion, osteoclast differentiation, and IL-17 signaling pathway, were enriched by the upregulated DEGs. Three categories (nicotine addiction, neuroactive ligand–receptor interaction, and cAMP signaling pathway) were due to downregulated DEGs. The other five categories (ECM–receptor interaction, cell adhesion molecules, PI3K-Akt signaling pathway, MAPK signaling pathway, and Rap1 signaling pathway) were co-regulated by the upregulated and downregulated DEGs (Figure 6B).

According to the comparison of the enriched KEGG categories of the total DEGs in IR24h vs. Sham with those of EA + IR24h vs. EASham, we found that there were 115 identical categories in these 2 groups. Except for the nicotine addiction pathway, which was exclusively enriched by the downregulated DEGs of the EA + IR24h group, the other top 20 categories of these 2 groups overlapped with these 115 categories. In addition, among the top 20 pathways of these 2 groups, 9 categories were the same, and 7 of them were attributed to the upregulated DEGs in both groups, including the TNF signaling pathway, NF-kappa B signaling pathway, cytokine–cytokine receptor interaction, osteoclast differentiation, IL-17 signaling pathway, chemokine signaling pathway, and hematopoietic cell lineage, which are typically inflammation response pathways. Furthermore, the other two common pathways, PI3K-Akt and MAPK signaling pathways, were attributed to the upregulated DEGs in the IR24h group. Still, in the EA + IR24h group, they were enriched in both upregulated and downregulated DEG categories (Supplementary Figures 3, 4 and Figures 6A,B).

### EA Pretreatment Attenuated the Changes of the Cerebral Transcriptome in Rats With I/R Injury

**FIGURE 7:**
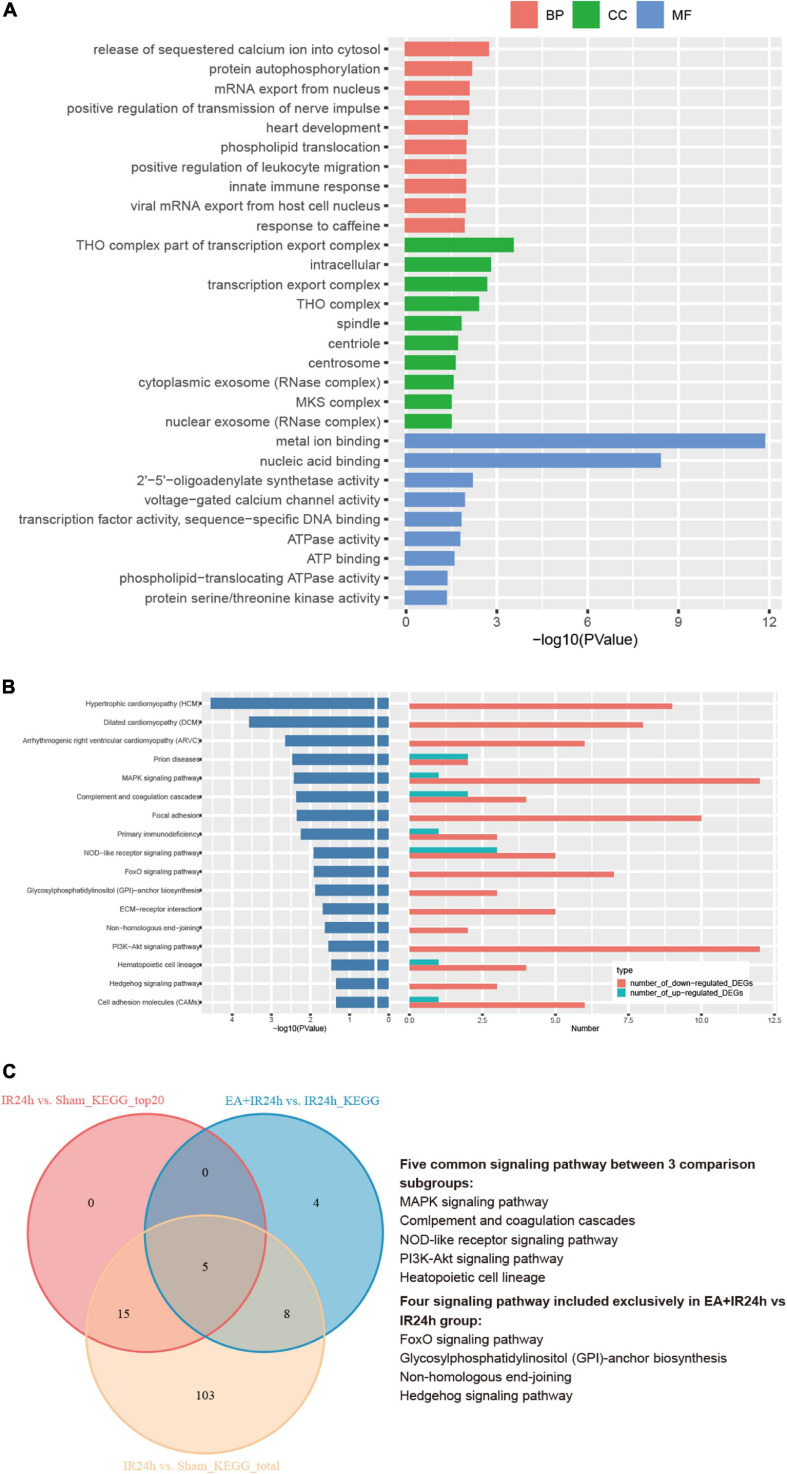
Bioinformation analysis of the differentially expressed genes (DEGs) in the EA + IR24h group vs. the IR24h group. **(A)** Top 10 gene ontology (GO) categories and corresponding –log10 (*P*-value). **(B)** Kyoto Encyclopedia of Genes and Genomes (KEGG) pathway analysis results of the DEGs, the top20 categories of KEGG signaling pathway and corresponding –log10 (*P*-value), and the corresponding numbers of up- and down-regulated DEGs in EA + IR24h vs. IR24h. **(C)** Venn diagram showing common and unique numbers of signaling pathway annotations for DEGs in EA + IR24h vs. IR24h.

To further decipher the underlying neuroprotective mechanism of EA pretreatment on I/R injury, the IR24h group was taken as a control to analyze the DEGs of the EA + IR24h group. The results showed that 48.7% of the DEGs in EA + IR24h vs. IR24h groups were upregulated and 51.3% were downregulated (Supplementary Table 6). And 20.7% of the downregulated DEGs in the EA + IR24h group belonged to the upregulated DEGs of the IR24h group, and 24.0% of the upregulated DEGs in the EA + IR24h group belonged to the downregulated DEGs of the IR24h group (Supplementary Figure 5). Corresponding to it, in the top 50 downregulated DEGs in the EA + IR24h group, 16 genes (32%) were the upregulated DEGs in the IR24h group, including mmp2, IL-17r, Tnfsf18, Tlr8, Lilrb4, and Plk4 (Supplementary Table 7). Meanwhile, among the top 50 upregulated DEGs in the EA + IR24h group, only 6 genes (12%) were downregulated by I/R, namely, Nat8f5, Dbp, Il16, Dcxr, Cartpt, and Hmgcs2 (Supplementary Table 8). To provide more detailed information, 498 DEGs (401 upregulated and 97 downregulated) with a threshold of |log2(FC)| > 1 and *Padj* < 0.05 were further subjected to bioinformatic analysis. GO annotation analysis revealed that these DEGs were mainly located in the THO complex, transcription export complex, intracellular, spindle, centrosome, cytoplasmic, nuclear exosome, and so on. They were primarily clustered in the molecular functions of metal ion binding, nuclear acid binding, 2′–5′-oligoadenylate synthetase activity, voltage-gated calcium channel activity, transcription factor activity, etc., and involved in multiple biological processes, such as the release of sequestered calcium ions into cytosol, protein autophosphorylation, viral mRNA export from host cell nucleus, innate immune response, positive regulation of transmission of nerve impulse, and heart development (Figure 7A). KEGG pathway analysis indicated that these DEGs were significantly enriched in 17 signaling pathway categories, including heart development-related pathways (e.g., hypertrophic cardiomyopathy, dilated cardiomyopathy, and arrhythmogenic right ventricular cardiomyopathy), cell apoptosis and inflammation response-related pathways (e.g., NOD-like receptor signaling pathway, MAPK signaling pathway, PI3K-Akt signaling pathway, FoxO signaling pathway, cell adhesion molecules, and ECM–receptor interaction), and immune-regulation-related pathways [e.g., primary immunodeficiency, complement and coagulation cascades, prion diseases, and glycosylphosphatidylinositol (GPI)-anchor biosynthesis] (Figure 7B). Comparing the KEGG results of the EA + IR24h and the IR24h groups, we surprisingly found that 13 out of 17 pathways in the EA + IR24h group were also enriched in the IR24h group. And 11 of these 13 categories were only attributed to I/R-induced upregulated DEGs. Moreover, 5 of these 11 categories, namely, the MAPK signaling pathway, NOD-like receptor signaling pathway, PI3K-Akt signaling pathway, complement and coagulation cascades, and hematopoietic cell lineage, were in the top 20 significantly signaling pathway categories of the IR24h group (Figure 7C). These results demonstrated that EA pretreatment could partially reverse I/R injury-induced abnormal gene expression profiles, particularly related to immuno-inflammation and stress signaling.

### Validation of the RNA-Seq Data

**FIGURE 8:**
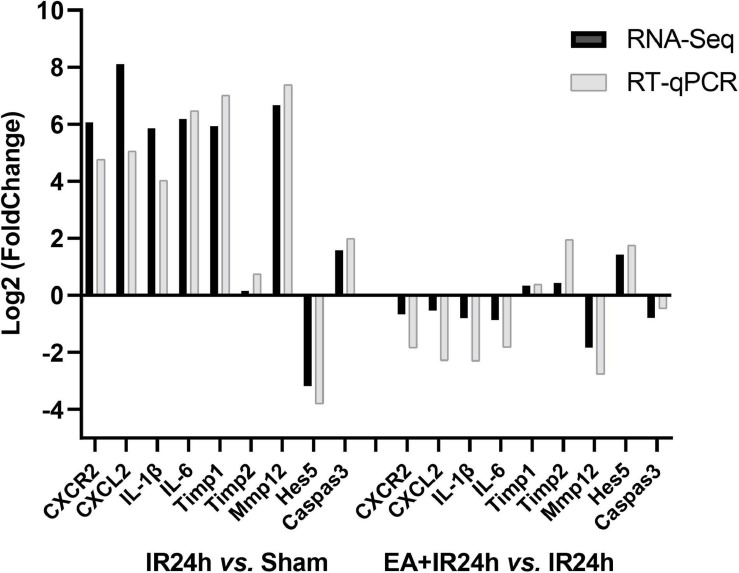
Quantitative real-time reverse transcription polymerase chain reaction (RT-qPCR) validation of RNA-Seq results. Data for comparison of fold change (log2) in differential expression values determined by RNA-Seq (black) and RT-qPCR (gray) for differentially expressed genes (DEGs) are shown.

To verify the RNA-Seq data, we applied the quantitative real-time reverse transcription-polymerase chain reaction (RT-qPCR) analysis to detect nine genes’ relative mRNA expressions. They were BBB integrity-related genes (*Timp1*, *Timp2*, and *Mmp12*), immune inflammation response genes (*CXCR2*, *CXCL1*, *IL*-1β, and *IL-6*), pro-apoptosis gene *caspase3*, and neurogenesis-related gene *Hes5*. The results demonstrated that I/R injury led to increased *CXCR2*, *CXCL1*, *IL-1*β, *IL-6*, *Mmp12*, *Timp1*, and *caspase3* mRNA expression and a decrease of *Hes5* but did not affect *Timp1*. Compared to that in the IR24h group, EA preconditioning significantly decreased the mRNA expression of *CXCR2*, *CXCL1*, *IL-1*β, *IL-6*, *Mmp12*, and *caspase3* and increased *Timp1*, *Timp2*, and *Hes5* mRNA expressions. These results were consistent with the RNA-Seq data, indicating the reliability of our RNA-Seq data (Figure 8).

## Discussion

The Yellow Emperor’s Classic of Internal Medicine, an essential classic of traditional Chinese medicine, mentioned that “The Sage treats a disease before its onset, deals with turmoil before it ferments.” It means that an excellent doctor often intervenes as early as possible to prevent the occurrence or spread of the disease. The oldest known document in Chinese medicine is, in fact, consistent with the modern medicine view that “prevention is more important than treatment.” As a part of traditional Chinese medicine, acupuncture has played an essential role in treating and preventing diseases over thousands of years because it is a practical, easily operated, economic therapy. Applying acupuncture in healthy or mildly sick patients to stimulate the meridians and enhance the body’s resistance to disease can prevent disease or reduce the extent of damage following illness (Wang and Liang, 2008).

The Baihui acupoint (GV20) is the most commonly applied acupuncture point in ischemia stroke research. The meridian theory in traditional Chinese medicine indicated that the Du meridian was closely related to the spinal cord, and GV20 is one of the acupoints of the Du meridian. Meanwhile, GV20 is in the projection area of the motor and sensory cortex and the projection area of the anterior cerebral artery in the neuroanatomy of western medicine (Li and Wang, 2013; Zhu et al., 2017). Many preclinical studies had shown that pretreatment of multiple or single acupuncture at GV20 has a neuroprotective effect on the stroke rat model (Xiong et al., 2003; Wang et al., 2005; Zhu et al., 2017). However, its underlying mechanism is still not well-elucidated. This study detected the histological and genome-wide transcription profile changes in healthy rat brains with EA treatment and the I/R injury brains after EA precondition. We confirm that EA pretreatment can significantly decrease the permeability of the BBB and infarction volume and improve the neurologic function of SD rats with I/R injury. At the same time, there was no histological change in the brain of the EA-pretreated healthy rat. Our result showed that preconditioning with EA could increase cerebral ischemic tolerance in rats, consistent with former research (Xiong et al., 2003; Wang et al., 2012; Long et al., 2019).

To explore the underlying mechanism of EA precondition-induced ischemic tolerance, we used RNA-Seq to generate gene expression profiles in the brains of EA-pretreated rats. The results showed that acupuncture precondition could significantly regulate 217 gene expressions in a healthy rat brain. Of the DEGs, 32.26% (70 out of 217) had expression changes that were more than twofold (Supplementary Table 6). According to the bioinformatic analysis, the protein products of these DEGs specifically formed two interacting function clusters. One is associated with protein digestion and absorption enriched by the downregulated DEGs, such as *Col6a1*, *Col11a*, *Col11a1*, *Col5a1*, *Col27a1*, and *Col24a1*. The reduction of these protein expressions might be attributed to the maintenance of BBB integrity in ischemic stroke. Another function cluster is associated with protective host defense, which was enriched by the upregulated DEGs. For instance, protein products of *Ifit1*, *Ifit12*, *Ifit13*, and *Ifit127* are interferon-induced proteins with tetratricopeptide repeats (IFITs), which have been reported to inhibit virus replication by binding and regulating the functions of cellular and viral proteins and RNAs. These proteins exerted critical roles in protecting the host from viral pathogenesis (Fensterl and Sen, 2015). *Oas1* and *Oasl2* belong to the 2′–5′-oligoadenylate synthetase (Oas) gene family, of which protein products are OAS-like (OASL) proteins, the crucial components of innate immunity in mammals. These proteins were identified to be strongly induced following viral infection through engaging the RNA sensor RIG-I and increasing signaling through this pathway to enhance the antiviral type I IFN response. In addition, OASL also responds to some cytosolic and vacuolar replicating bacterial pathogens, which showed that OASL played an essential role in the innate immune response to infection with a variety of pathogens (Perelygin et al., 2006; Leisching et al., 2017). Stimulated by alpha/beta IFN, *Mx1* and *Mx2* proteins participated in host antiviral defense systems involving IFNs and IFN-induced antiviral proteins (Meier et al., 1990). The central nervous system has always been regarded as an immune privilege place. However, in recent years, more and more data suggested that the meninges around the brain and spinal cord contain a diverse population of innate and adaptive immune cells, which provide monitoring and protection of the brain along with the brain barrier structure under homeostasis conditions and various neurological disorders (Mrdjen et al., 2018; Van Hove et al., 2019; Ringstad and Eide, 2020). Zhang et al. (2018) reported the transient-opening effect of EA precondition on BBB in the healthy rat. Together with these researches, we postulated that activating anti-infection immunity and restoring disruption of extracellular matrix proteins or collagens might be associated with the entrance of large molecules into the blood due to the minor non-pathogenic EA precondition-induced short-time opening of BBB, forming EA-precondition-induced ischemic tolerance.

We subsequently compared the characteristics of post-stroke transcriptome profiles in the healthy rat with EA treatment and the EA-preconditioned rat under subsequent I/R injury. There were many similarities in transcriptome responses on I/R damage for non-pretreated and EA-pretreated rats. For the DEGs, I/R damage induced 4,798 and 5,881 DEGs, respectively, in IR24h vs. Sham and EA + IR24h vs. EASham, in which about 35% of DEGs had an expression change that was more than twofold. And the number of upregulated genes was greater than that of downregulated genes (Supplementary Table 6), which is consistent with the previous research (Dergunova et al., 2018). Then, we analyzed these two sets of DEGs with |log2(FC)| > 1. A total of 1,124 DEGs (about 68.6% in the IR24h group and 53.5% in the EA + IR24h group) were the same, 74.6% of which were upregulated. These upregulated genes were predominantly encoding inflammatory, immune, and stress response-related proteins, such as chemokines (*Cxcl2*, *Cxcl1*, *Ccl2*, *Ccl3*, and *Cxcr2*), cytokines (*Il6*, *Il1b*, *Il12b*, and *Tnfs18*), heat-shock proteins (*Hspb1* and *Hspa1b*), immunoprotein (*Oscar*, *DIgR1*, *F7*, *Cd207*), and others (*Mmp12*, *Timp1*, *Runx3*, and *Kng1*) (Supplementary Table 3). Bioinformatic analysis results also showed a significant proportion (about 61–89%) of GO and KEGG enrichment categories were the same, especially in the top significant categories in these two comparative groups. For example, among the top 10 significant categories of cell components, 9 categories were the same (Figure 5). Meanwhile, 86.7% functional categories and 88.9% KEGG pathways in the IR24h group were overlapped with that of the EA + IR24h group (Table 1 and Supplementary Figure 3). The identical function categories of these two comparative groups were mainly involved in glycoprotein, immunity, inflammatory response, cell adhesion, apoptosis, etc. The top 20 overlapped KEGG pathways, including TNF signaling pathway, NF-kappa B signaling pathway, cytokine–cytokine receptor interaction, IL-17 signaling pathway, and chemokine signaling pathway, are associated with the immuno-inflammation response, which was consistent with previous studies conducted by Dergunova et al. (2018) and Wang et al. (2017) (Table 1 and Supplementary Figure 4). Furthermore, based on KEGG pathway enrichment analysis, we also confirmed that the upregulated DEGs in the post-stroke brain played an essential role in the response of brain cells to I/R injury. We found that in both IR24h vs. Sham and EA + IR24h vs. EASham, the upregulated DEG enriched categories were obviously more than that of the downregulated DEGs, and over 90% of upregulated DEG enriched categories were overlapped with that of the total DEGs. Concurrently, 74.8% of total DEG enriched categories in the IR24h group and 60.5% total DEG enriched categories in the EA + IR24h group exclusively contributed to the upregulated DEGs. In particular, in the top 20 total DEG enriched pathways of the IR24h group, 19 were overlapped with the upregulated DEGs. In contrast, exclusively enriched ratios in the downregulated DEGs were only 16.8 and 28.6%, respectively. Even in the top 50 categories, only 1 category coincided with the downregulated DEGs (Supplementary Figures 1, 2).

Although the expression profiles of non-pretreated and EA-pretreated rats were somewhat similar, there were still some discrepancies between them. Firstly, the gene expression pattern of non-common regulated DEGs in EA + IR24h vs. EASham was contrary to the above results. The upregulated DEG number was less than that of the downregulated DEGs (Figure 4B). Secondly, for the common genes in the top 50 upregulated DEGs of these 2 comparative groups, most of the upregulated DEGs’ expression fold change in EA-pretreated rats was less than that of non-pretreated rats. However, the downregulated common DEG expression change fold of EA-pretreated rats was more than that of non-pretreated rats (Supplementary Table 3). Thirdly, functional analysis results indicated that more downregulated DEGs in EA-pretreated stroke rats participated in the 26 common function categories, and these downregulated DEGs encoding proteins were also involved in the neuro-excitotoxicity-related processes such as ion channel and transport (ligand-gated ion channel, chloride channel, voltage-gated channel, and symport), postsynaptic cell membrane, and synapse. Meanwhile, the upregulated DEGs encoding proteins were involved in adaptive immunity, cell junction, integrin, and the growth factor binding process, which were associated with BBB integrity and cell survival (Table 1). Lastly, compared with the non-pretreated stroke rat, downregulated DEG enriched pathways in the EA-pretreated stroke rat had a higher ratio than that in the total DEG enriched pathways. Among the top 20 significant enriched pathways of all DEGs, 3 pathways in the EA + IR24h group overlapped with the downregulated DEGs. In the IR24h group, there was no overlapping with downregulated DEGs (Supplementary Figures 1, 2). These differences indicated that the downregulated DEGs in the EA-pretreated stroke rats played a more critical role in the pathological process of cerebral I/R injury than in non-pretreated rats. It might contribute to the difference in pathological damage between EA-pretreated and non-pretreated rats during I/R injury.

Therefore, we took the IR24h group as a control to further decipher the mechanism of the EA pretreatment’s neuroprotective effect on stroke injury. Over 20% of I/R injury-induced upregulated or downregulated genes were reversed or ameliorated by EA pretreatment. For instance, among the top 50 downregulated DEGs of the EA + IR24h group, 14 genes belonged to the top 50 upregulated DEGs of the IR24h group, such as *Tnfsf18*, *Mmp12*, *Il17r*, and *Tlr8* (Supplementary Figure 5 and Supplementary Table 7). In addition, in the EA + IR24h group, the number of downregulated DEGs was more than that of upregulated DEGs, especially in the DEGs with |log2(FC)| > 1, in which 80.5% were downregulated (Supplementary Table 6 and Figure 2B). Bioinformatic analysis also showed that, apart from the prion disease, NOD-like receptor signaling pathway, and complement and coagulation cascades pathway, the other 14 KEGG pathways enriched by all DEGs with |log2(FC)| > 1 in the EA + IR24h group were all overlapped with the downregulated DEG enriched pathways. More importantly, in these 14 pathway categories, 10 categories overlapped with I/R injury-induced upregulated DEG enriched pathways, including the MAPK signaling pathway, PI3K-Akt signaling pathway, focal adhesion, primary immunodeficiency, ECM–receptor interaction, and cell adhesion molecules. They were reported to be associated with apoptosis, inflammation, and immune response and played essential roles in I/R injury-induced cascade reactions. These results indicated that EA pretreatment could directly inhibit partial I/R injury-induced pathological signaling pathways, particularly the immuno-inflammation-related pathways, to exert its neuroprotective effect on the stroke rat model. Finally, we note that there were 7 common upregulated genes (*Irf7*, *Oas1a*, *Rtp4*, *Oas1b*, *Usp18*, *Ifi27*, and *Isg15*) and 5 downregulated genes (*Plekhn1*, *Lime1*, *Nfxl1*, *F8*, and *Ints6l*) in the top 50 DEGs of both the EA + IR24h group and the EASham group (Supplementary Figure 5). As mentioned above, these genes were mainly involved in antivirus and anti-inflammation immune response processes. It indicated that these EA-pretreatment-activated immune and stress response genes in healthy rats contribute to its anti-ischemic injury effect under subsequent I/R injury.

Collectively, EA pretreatment could stimulate central anti-inflammation immune response-associated signaling pathways on the healthy rats and limit neuro-immune inflammation-induced cascade by subsequent I/R injury. It suggested that acupuncture is a promising preventive approach for improving ischemic tolerance for stroke.

## Conclusion

Electroacupuncture preconditioning at GV20 could activate the brain immune response of healthy rats and improve ischemic tolerance of SD rats under I/R injury. It could partially reverse or ameliorate I/R injury-related gene expression changes, especially genes participating in the immune and inflammation responses. For the first time, this study provides informative genome-wide profiles of gene expression in EA-preconditioned SD rats under subsequent I/R injury, as well as healthy rats with EA treatment. It reveals the potential mechanisms of acupuncture precondition and provides new strategies and targets for preventing ischemic stroke.

## Data Availability Statement

The data presented in the study are deposited in the SRA repository, accession number is PRJAN748567.

## Ethics Statement

The animal study was reviewed and approved by the Institutional Animal Care Unit Committee of the Nanjing University of Chinese Medicine.

## Author Contributions

SF and SL initiated the research and designed the experiments. MY, XL, YW, LM, YQ, and YC conducted the experiments. XJ, HX, CL, and QL performed the bioinformatic and statistical analyses. SF, QL, and SL wrote and revised the manuscript. All authors read and approved the final manuscript.

## Conflict of Interest

The authors declare that the research was conducted in the absence of any commercial or financial relationships that could be construed as a potential conflict of interest.

## Publisher’s Note

All claims expressed in this article are solely those of the authors and do not necessarily represent those of their affiliated organizations, or those of the publisher, the editors and the reviewers. Any product that may be evaluated in this article, or claim that may be made by its manufacturer, is not guaranteed or endorsed by the publisher.
