# Supplementary material for: Genome-Wide Transcription Analysis of Electroacupuncture Precondition-Induced Ischemic Tolerance on SD Rat With Ischemia–Reperfusion Injury
Source: Front Genet. 2021 Aug 16;12:719201. doi: 10.3389/fgene.2021.719201 (PMC8415365; doi:10.3389/fgene.2021.719201)
Supplement: Supplementary file 1 [file Data_Sheet_1.PDF]

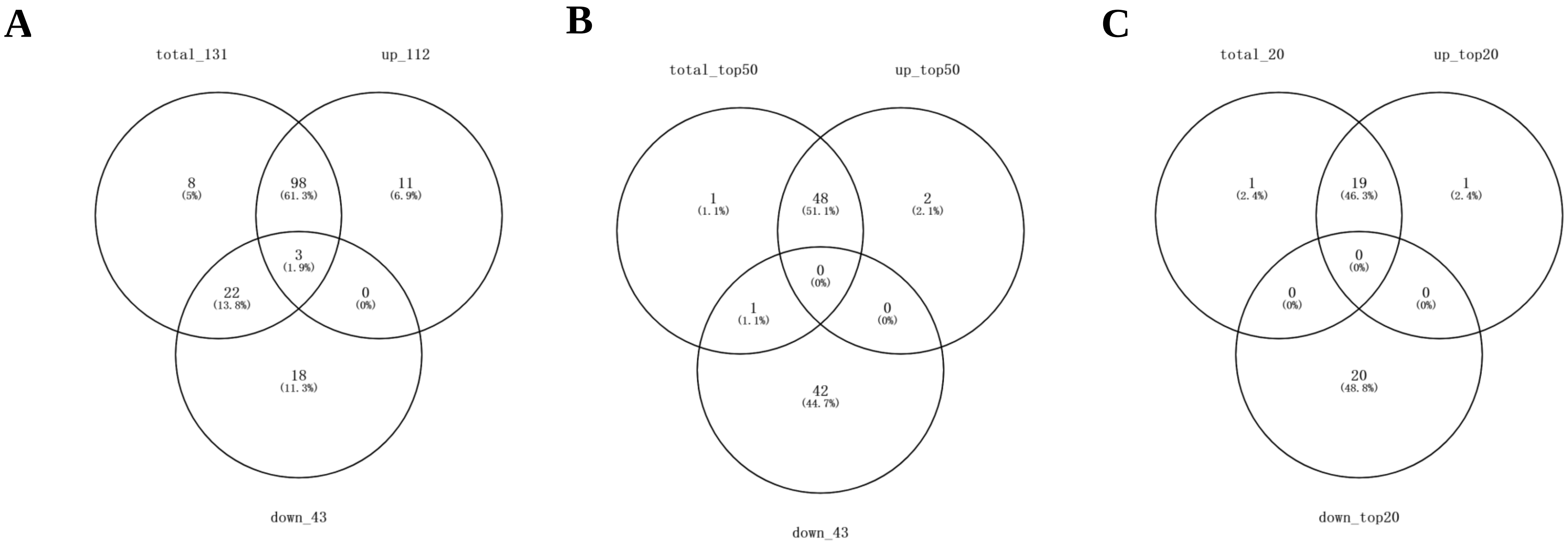

Supplementary Figure 1. Venn diagrams result of KEGG pathways in IR24h *vs.* Sham. **(A)** Common and unique numbers of signaling pathway annotations for total DEGs、up-regulated DEGs、down-regulated DEGs in IR24h *vs.* Sham. **(B)** Common and unique numbers of the top 50 signaling pathway annotations for total DEGs、up-regulated DEGs、down-regulated DEGs in IR24h *vs.* Sham. **(C)** Common and unique numbers of the top 20 signaling pathway annotations for total DEGs、up-regulated DEGs、down-regulated DEGs in IR24h *vs.* Sham.

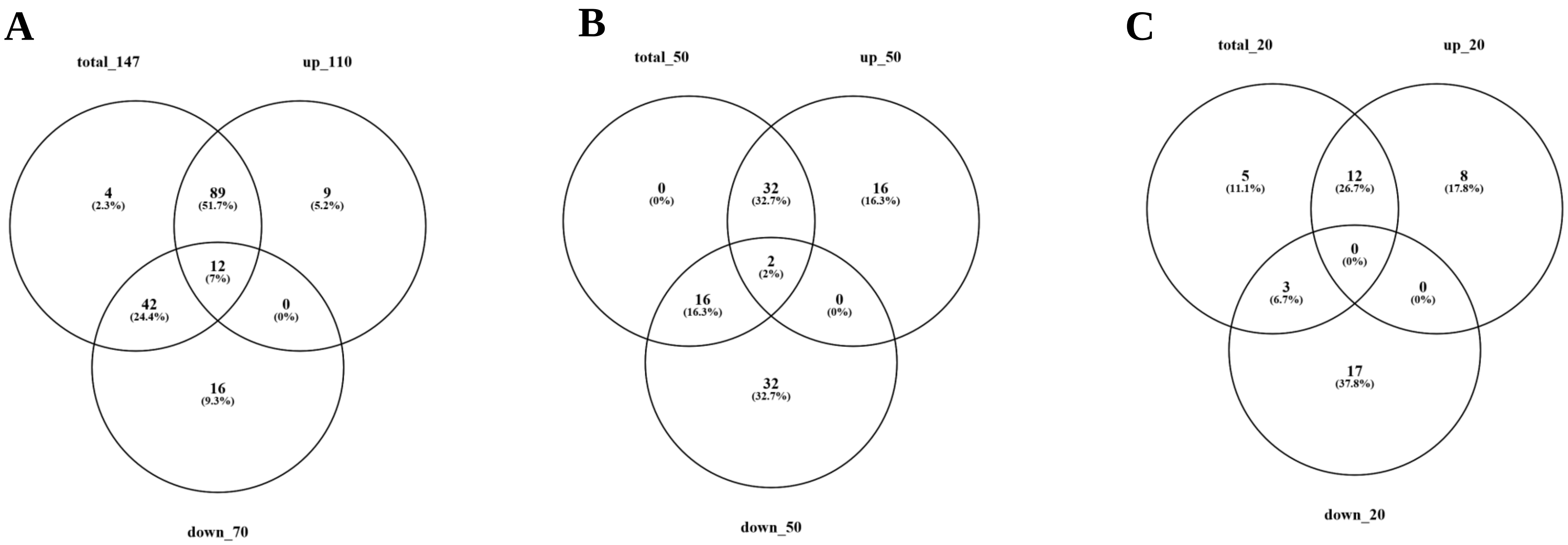

Supplementary Figure 2. Venn diagrams result of KEGG pathways in EA+IR24h *vs.* EAsham. **(A)** Common and unique numbers of signaling pathway annotations for total DEGs、up-regulated DEGs、down-regulated DEGs in EA+IR24h *vs.* EAsham. **(B)** Common and unique numbers of the top 50 signaling pathway annotations for total DEGs、up-regulated DEGs、down-regulated DEGs in EA+IR24h *vs.* EAsham. **(C)** Common and unique numbers of the top 20 signaling pathway annotations for total DEGs、up-regulated DEGs、down-regulated DEGs in EA+IR24h *vs.* EAsham.

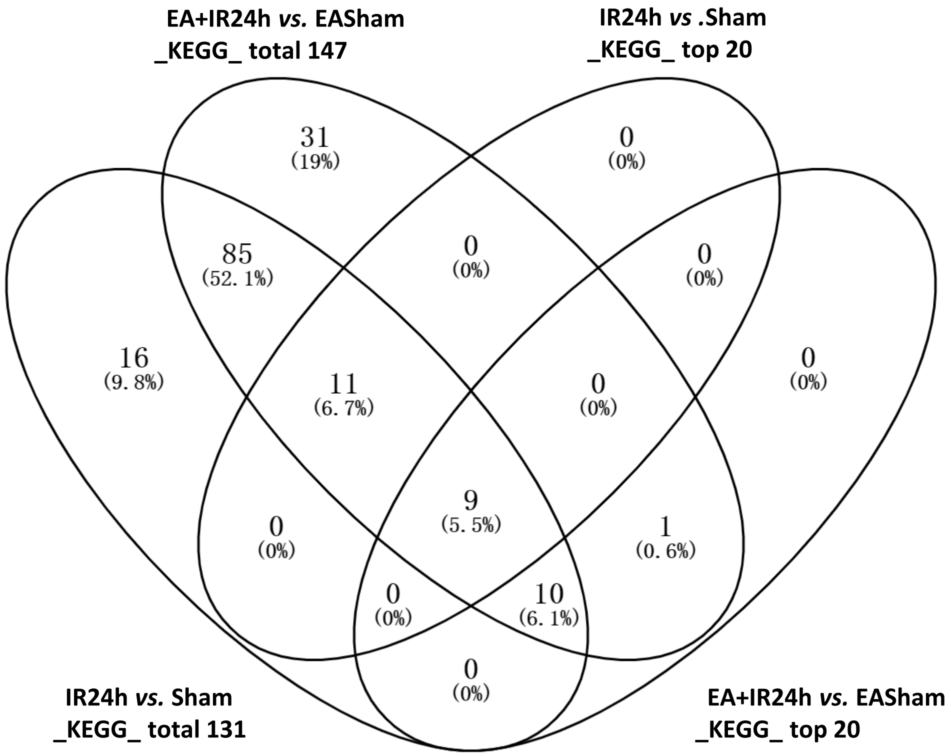

Supplementary Figure 3. Comparison results of KEGG pathway of EA pretreatment and non-pretreatment rat with subsequent I/R injury. Venn diagrams showed the common and unique numbers of total and the top 20 signaling pathway annotations between IR24h *vs.* Sham and EA_+IR24h *vs.* EASham.

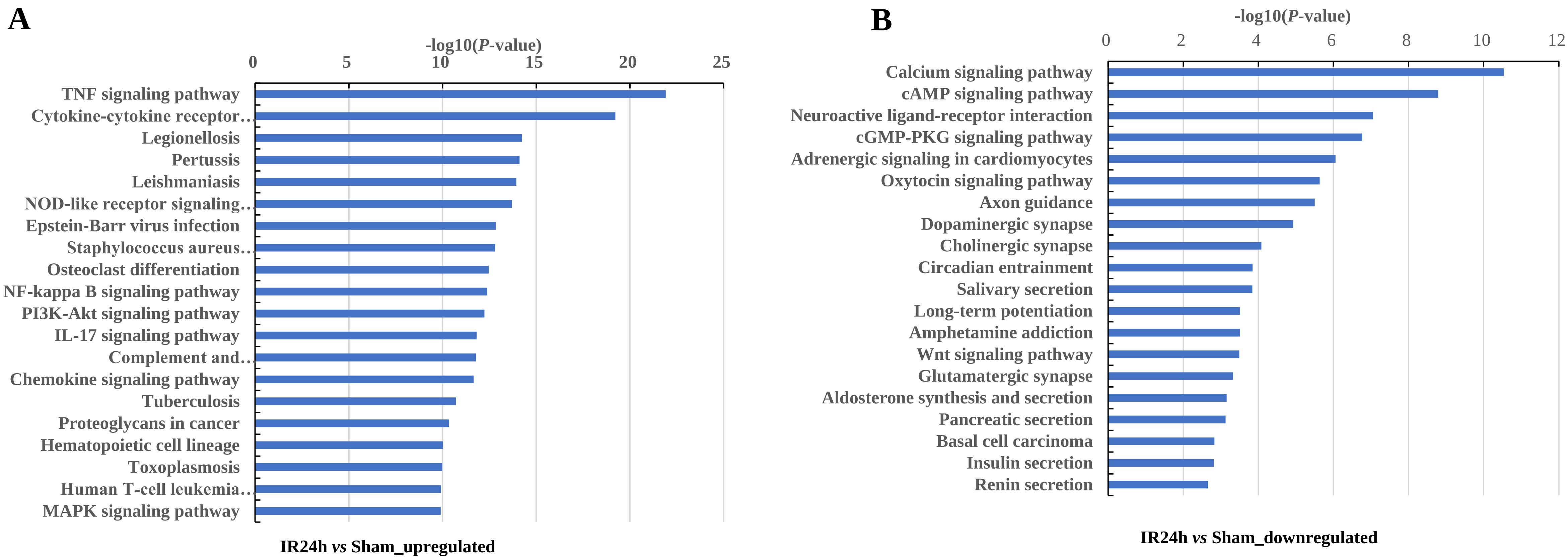

A
-log10(P-value)
0
5
10
15
20
25
TNF signaling pathway
Cytokine-cytokine receptor…
Legionellosis
Pertussis
Leishmaniasis
NOD-like receptor signaling…
Epstein-Barr virus infection
Staphylococcus aureus…
Osteoclast differentiation
NF-kappa B signaling pathway
PI3K-Akt signaling pathway
IL-17 signaling pathway
Complement and…
Chemokine signaling pathway
Tuberculosis
Proteoglycans in cancer
Hematopoietic cell lineage
Toxoplasmosis
Human T-cell leukemia…
MAPK signaling pathway
IR24h vs Sham_upregulated
B
-log10(P-value)
0
2
4
6
8
10
12
Calcium signaling pathway
cAMP signaling pathway
Neuroactive ligand-receptor interaction
cGMP-PKG signaling pathway
Adrenergic signaling in cardiomyocytes
Oxytocin signaling pathway
Axon guidance
Dopaminergic synapse
Cholinergic synapse
Circadian entrainment
Salivary secretion
Long-term potentiation
Amphetamine addiction
Wnt signaling pathway
Glutamatergic synapse
Aldosterone synthesis and secretion
Pancreatic secretion
Basal cell carcinoma
Insulin secretion
Renin secretion
IR24h vs Sham_downregulated

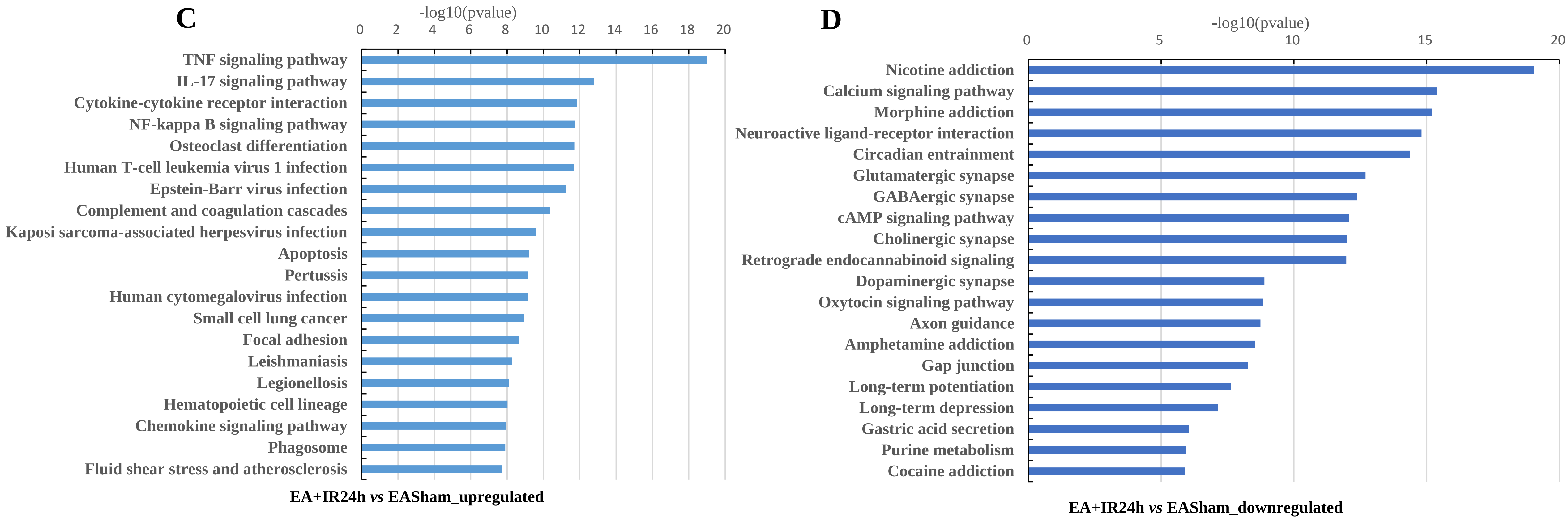

Supplementary Figure 4. KEGG pathway analysis of the up-and down-regulated DEGs in EA pretreatment and non-pretreatment rats with subsequent I/R injury (A) Top 20 categories of KEGG signaling pathway and corresponding *P*-values for the up-regulated DEGs in **IR24h *vs.* Sham.** (B) Top 20 categories of KEGG signaling pathway and corresponding *P*-values for the down-regulated DEGs in **IR24h *vs.* Sham.** (C) Top 20 categories of KEGG signaling pathway and corresponding *P*-values for the up-regulated DEGs in **EA+IR24h *vs*. EASham.** (D) Top 20 categories of KEGG signaling pathway and corresponding *P*-values for the down-regulated DEGs in **EA+IR24h *vs*. EASham**

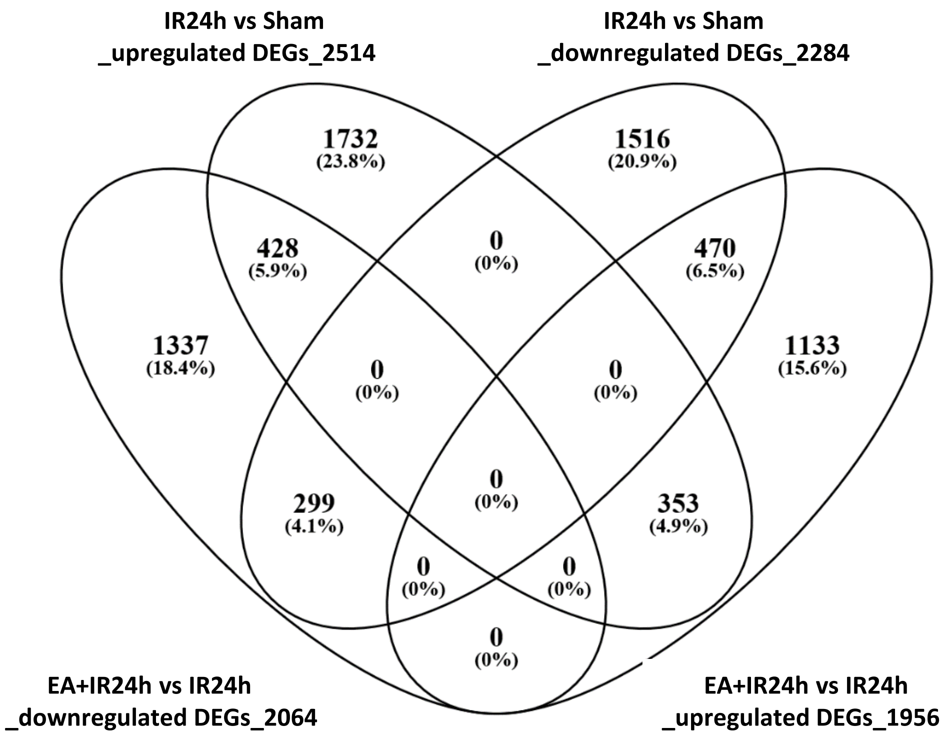

Supplementary Figure 5. DEGs Comparison results of the EA+IR24 group *vs.* the IR24 group Venn diagrams showed the common and unique numbers for the up- and down_regulated DEGs in EA+IR24 group vs. the IR24 and the up_ and down_regulated DEGs in IR24h vs Sham.

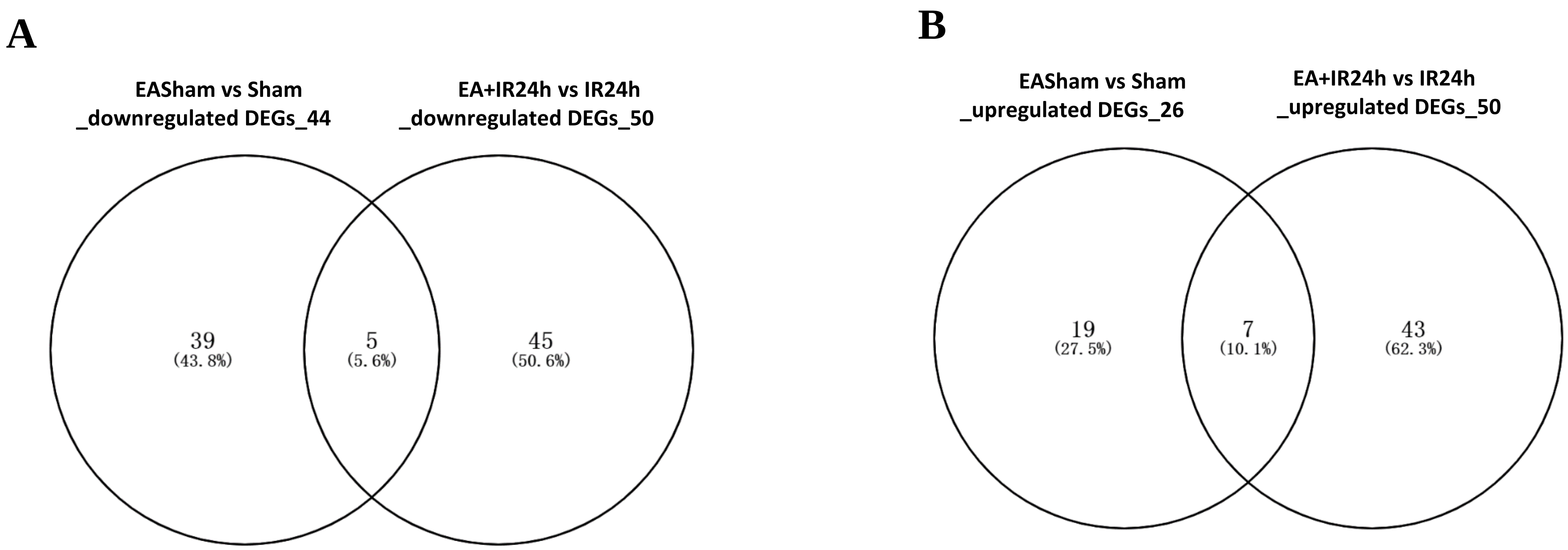

Supplementary Figure 6. DEGs Comparison results of the EA+IR24 group *vs.* the EASham group Venn diagrams showed the common and unique numbers for the top 50 up- and down_regulated DEGs in EA+IR24 group vs. the IR24 and the up_ and down_regulated DEGs in EASham vs. Sham.
